# Comparative analysis of 12 water lily plastid genomes reveals genomic divergence and evolutionary relationships in early flowering plants

**DOI:** 10.1007/s42995-024-00242-0

**Published:** 2024-08-15

**Authors:** Weicai Song, Wenbo Shi, Huan Wang, Zirui Zhang, Ruiqing Tao, Jin Liu, Shuo Wang, Michael S. Engel, Chao Shi

**Affiliations:** 1 College of Marine Science and Biological Engineering, Qingdao University of Science and Technology, Qingdao, 266042 China; 2 Plant Germplasm and Genomics Center, Germplasm Bank of Wild Species in Southwest China, Kunming Institute of Botany, The Chinese Academy of Sciences, Kunming, 650204 China; 3 Yunnan Institute of Tropical Crops, Xishuangbanna, 666100 China; 4 American Museum of Natural History, New York, NY 10024-5192 USA; 5 Natural History Museum, and Department of Ecology & Evolutionary Biology, University of Kansas, Lawrence, KS 66045 USA

**Keywords:** Aquatic plants, *Nymphaea*, Phylogenetic analysis, Plastid genomics, Structural variation

## Abstract

The aquatic plant *Nymphaea*, a model genus of the early flowering plant lineage Nymphaeales and family Nymphaeaceae, has been extensively studied. However, the availability of chloroplast genome data for this genus is incomplete, and phylogenetic relationships within the order Nymphaeales remain controversial. In this study, 12 chloroplast genomes of *Nymphaea* were assembled and analyzed for the first time. These genomes were 158,290–160,042 bp in size and contained 113 non-repeat genes, including 79 protein-coding genes, 30 tRNA genes, and four rRNA genes. We also report on codon usage, RNA editing sites, microsatellite structures, and new repetitive sequences in this genus. Comparative genomics revealed that expansion and contraction of IR regions can lead to changes in the gene numbers. Additionally, it was observed that the highly variable regions of the chloroplast genome were mainly located in intergenic regions. Furthermore, the phylogenetic tree showed the order Nymphaeales was divided into three families, and the genus *Nymphaea* can be divided into five (or three) subgenera, with the subgenus *Nymphaea* being the oldest. The divergence times of nymphaealean taxa were analyzed, with origins of the order Nymphaeales and family Nymphaeaceae being about 194 and 131 million years, respectively. The results of the phylogenetic analysis and estimated divergence times will be useful for future evolutionary studies of basal angiosperm lineages.

**Supplementary Information:**

The online version contains supplementary material available at 10.1007/s42995-024-00242-0.

## Introduction

Angiosperms are plants that bear flowers and fruits that encase seeds are the most diverse land plants with about 300,000 species (Singh and Singh 2022). The basal angiosperms are essential for studying the origin and evolution of flowering plants (Xi et al. 2014). They contain some ancestral morphological and structural features that have attracted extensive interest from botanists (Yamada et al. 2001). Studying basal angiosperms has profound implications for understanding angiosperm diversity, adaptation, genome evolution, and development (Li et al. 2021a; Qiu et al. 1999). Nymphaeales is the second-most basal lineage after Amborellales (Soltis et al. 2000), and plays an important role in the study of the origin of early angiosperm lineages (Zanis et al. 2002). Although it is now generally thought that the order Nymphaeales is divided into three families, namely Nymphaeaceae, Cabombaceae, and Hydatellaceae (Gruenstaeudl et al. 2017; Saarela et al. 2007), the phylogenetic relationships within Nymphaeales were controversial (He et al. 2018; Sun et al. 2021).

*Nymphaea* is a perennial aquatic herbaceous plant, commonly known as the water lily. It is the largest and most widely distributed genus in the Nymphaeaceae with approximately 45–50 species (Borsch et al. 2007), and not only provides food and habitat for aquatic animals, but also reduces water turbidity and stabilizes stream sediments in shallow freshwater ecosystems, both tropical and temperate (Dalziell et al. 2020; Parveen et al. 2022). In addition, most water lilies have considerable ornamental value (Borsch et al. 2007) and many are well-known because of their variety of flower colors, long flowering period and strong adaptability (Bhandarkar and Khan 2004). In addition to being ornamental, water lilies are added as ingredients in numerous products, including cosmetics, soaps, perfumes and traditional medicines (Bhandarkar and Khan 2004). Several kinds of water lilies are also used for sewage purification (Lavid et al. 2001; Lu et al. 2012). The genus *Nymphaea* is phenotypically diverse and exhibits high levels of inter-specific polymorphism (Heslop-Harrison 1955). Although various molecular marker methods such as inter-simple sequence repeats (ISSR), amplified fragment length polymorphism (AFLP), and random amplified polymorphic DNA (RAPD) have been applied to study aquatic plant evolution (Hu et al. 2012; Koga et al. 2007; Kumar et al. 2016), the diversity and structure of the *Nymphaea* genome should be further explored.

As an important genetic element in plant cells, the chloroplast genome deserves to be studied in depth because of its diversity (Brunkard et al. 2015). The DNA of higher plant chloroplasts is a double-stranded covalently closed circular molecule whose length varies among species. It is composed of four sequences, including two inverted repeats (IRa and IRb), one long single copy sequence (LSC), and one short single copy (SSC) sequence (Odintsova and Yurina 2003). Chloroplast genomes have been widely used in the study of molecular evolution and phylogeny due to their moderate size for sequencing and the good collinearity among chloroplast genomes of different plant groups for comparative analysis (Olmstead and Palmer 1994). Since the first complete plastid genome was applied to plant phylogeny, plastid phylogenomics has been widely used to resolve phylogenetic relationships in photosynthetic eukaryotes, including green algae (Sun et al. 2016) and land plants (Ruhfel et al. 2014). The increased availability of complete plastid genomes has considerably increased the genomic resources for analyses of evolutionary relationships, including those that are controversial such as among the early-diverging angiosperms (Gruenstaeudl et al. 2017).

In this study, the chloroplast genomes of 12 *Nymphaea* species were sequenced and assembled for the first time and submitted to the National Center for Biotechnology Information (NCBI). We analyzed these sequences to further reveal genomic differentiation and interspecific evolutionary relationships in early flowering plants by: (1) comparing the structure and content of these genomes; (2) analyzing their relative synonymous codon usage (RSCU) and RNA editing sites; (3) examining the pattern of repeat sequences and microsatellites; (4) comparing genomic variation levels; (5) identifying highly variable loci that are suitable as molecular markers; (6) evaluating the phylogenetic relationships of these and other closely related species; (7) estimating their divergence times. These results not only enriched the genetic information of *Nymphaea* for the utilization of germplasm resources but also clarified the phylogenetic relationships among basal angiosperm lineages.

## Material and methods

### Data preprocessing

All plastid genomes sequenced for this investigation were obtained from the NCBI public database (Supplementary Table S1) (Gruenstaeudl et al. 2017). Approximately 2.4 GB of high-quality raw reads for each species were obtained and used to assemble the complete chloroplast genome of *Nymphaea*. Raw data were pre-processed using Trimmomatic v0.39 software (Bolger et al. 2014), including the removal of adapter sequences and other special sequences from sequencing, such as reads with many ‘N’ bases. The quality of newly produced clean short reads was evaluated using FASTQC v0.11.9 (Andrews 2014) and MULTIQC v.1.7 (Ewels et al. 2016) software to select reads with Phred scores averaging above 35.

### Assembly and annotation of chloroplast genomes

We used two types of genome assembly. First, de novo splicing software GetOrganelle v 1.7.4 (Jin et al. 2020) was used to assemble the chloroplast genome sequence from the clean reads. Second, chloroplast-like (cp) fragments were selected from clean reads by comparing them to the reference sequence of *Nymphaea colorata* (MT107631) using BLAST (Camacho et al. 2009). The reads were assembled with SPAdes v 3.12.0 (Bankevich et al. 2012) by setting k-mer values as 35, 44, 71, and 101. The coverage of each sample was measured using Geneious software (Supplementary Fig. S1). The new complete chloroplast genome was annotated by the combined results from Plastid Genome Annotator (PGA) (Qu et al. 2019) and GeSeq v1.42 (Tillich et al. 2017). RNAmmer v.1.2 (Lagesen et al. 2007) and tRNAscan-SE v 1.21 (Lowe and Eddy 1996) were used to validate RNA genes with default settings. As a final check, GB2Sequin (Lehwark and Greiner 2019) was used to manually check the boundaries of introns or exons and the positions of start or stop codons, with the reference sequence in the GenBank database. Chloroplot (Zheng et al. 2020) was used to draw the circular map of the genomes. The 12 new chloroplast genomes of *Nymphaea* have been deposited in GenBank under accession numbers MW802262–MW802273.

### Sequence structure analysis

Relative synonymous codon usage was calculated by the Computer Codon Usage Bias function in MEGA 7 (Kumar et al. 2016). Information regarding genome length, GC content, and gene number of each region in the chloroplast genomes was obtained using Geneious Prime software (Kearse et al. 2012). A microsatellite identification tool (Beier et al. 2017) was used to find Simple Sequence Repeats (SSRs). The minimum number of repeats for motif lengths of 1, 2, 3, 4, 5, and 6 were 10, 4, 4, 3, 3, and 3, respectively. REPuter (Kurtz et al. 2001) was used to calculate four types of repeats with the following parameter settings: (1) Hamming distance of 3; (2) sequence identity higher than 90%; and (3) minimum repeat size of 20 bp. Predictive RNA Editor for Plants (PREP) was used to examine RNA editing sites from protein-coding sequences (CDS) with a cutoff value set to 0.8.

### Comparative analysis

The IRscope tool (Amiryousefi et al. 2018) was used to detect and visualize the contraction and expansion sequence of inverted repeats of the *Nymphaea* chloroplast genomes. To compare chloroplast genomic differences between species, sequence comparisons were performed on the online comparison tool mVISTA (Frazer et al. 2004), setting up the Shuffle-LAGAN (Brudno et al. 2003) method, and selecting *N. lotus* as the reference species. We also used DNAsp v6 software (Rozas et al. 2017) to calculate the nucleotide diversity (Pi) with 1000 bp window length and 50 bp step size KaKs_Calculator v2.0 (Wang et al. 2008) was used to calculate the ratio of non-synonymous mutation and synonymous mutation.

### Phylogenetic analysis and divergence time estimation

To construct the phylogenetic relationships of basal angiosperms, we selected 42 species of Nymphaeales and the basal angiosperm *Amborella trichopoda* (Yang et al. 2020) as outgroups (Supplementary Table S2). Two types of tree were constructed using the complete chloroplast genome sequence data as well as 84 protein-coding genes to frame the maximum likelihood (ML) topologies, respectively. MAFFT-X (Katoh and Standley 2013) was used to perform the multiple gene alignment MrBayes v3.1.2 (Ronquist and Huelsenbeck 2003) was used to conduct Bayesian inference (BI) analyses. Modeltest v3.7 (Posada and Buckley 2004) was used to find the best models by the Akaike information criterion (Posada and Crandall 1998). The GTR + G + I model was selected to construct the ML tree in MEGA X (Kumar et al. 2018), with the bootstrap value set to 1000. Well-supported clades were defined by having a posterior probability above 0.95 (PPBI > 0.95) and a bootstrap value above 70% (BSML > 70%) (Alfaro and Holder 2006).

To estimate the evolutionary timescale of Nymphaeales, we calibrated a relaxed molecular clock using one molecular dating and four fossil-based ages throughout the tree (more fossils are shown in Sect. “Phylogenetic analysis and divergence time estimation of Nymphaeales”). Gblocks v0.91 (Castresana 2000) was used to remove all gap positions, most variable sites, and ambiguous sites in the multiple alignments. The base mutation rates were calculated using BASEML from the PAML v4.8 package (Yang 2007). The divergence times were calculated using mcmctree (Puttick 2019), with samples drawn every 400 steps over a total of 10 million following the burn-in of 3 million steps. We checked for convergence and sufficient sampling by running the analysis in duplicate. Finally, Tracer v1.7.2 (Rambaut et al. 2018) was used to verify that the effective sample size value was above 200.

## Results

### General characteristics analysis

**Fig. 1:**
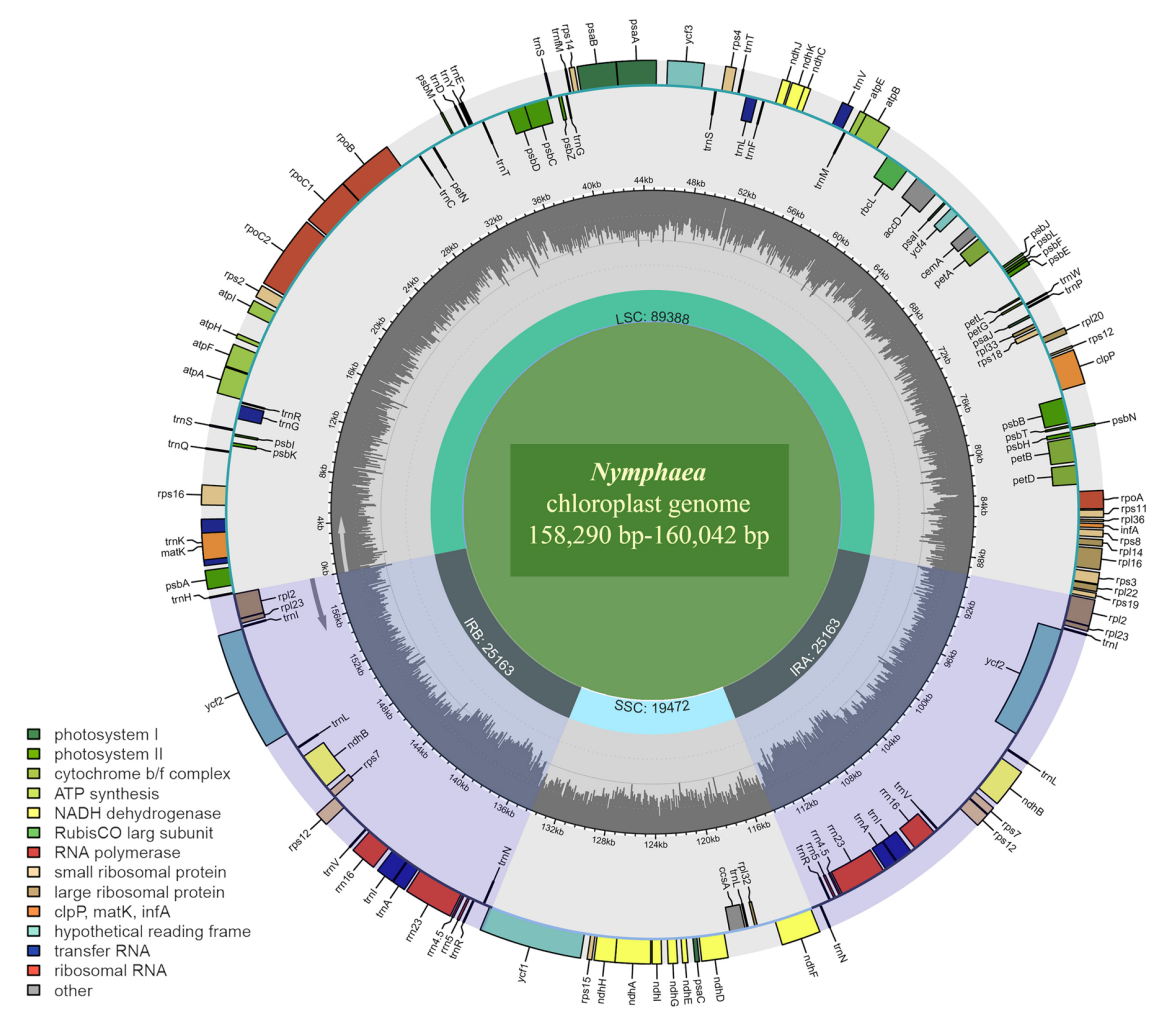
Circular maps of *Nymphaea amazonum*. The various functional groups are depicted by the colored bars. Small single-copy regions (SSC) and large single-copy regions (LSC) of the genome are separated by the range of inverted repeats (IRa and IRb), which are represented by dark areas. Genes drawn outside the circle are transcribed counterclockwise, whereas genes drawn inside the circle are transcribed clockwise. AT content is represented by the light gray inner circle, while GC content is represented by the dark gray inner circle

The complete chloroplast genome of *Nymphaea* had a typically circular structure consisting of four sequences, including a large single copy (LSC), a small single copy (SSC), and two inverted repeats (IRa/IRb) (Fig. 1). The genome size of the 12 species of *Nymphaea* varied from 158,290 to 160,042 bp, among which *N. immutabilis* was the largest (160,042 bp) and *N. tenerinervia* was the smallest (158,290 bp). The total GC content varied between 39.1% and 39.2% (Table 1), this great similarity indicating their high degree of affinity. The LSC region ranged from 88,499 bp (*N. tenerinervia*) to 90,118 bp (*N. immutabilis*) in size, and its total GC content was around 37.8%. The SSC region was between 19,283 bp (*N. rudgeana*) and 19,594 bp (*N. immutabilis*), and the total GC content ranged between 34.2% and 34.4%. The IR regions were between 50,300 bp (*N.* sp. NY590) and 50,396 bp (*N. gracilis*), and their GC content was about 43.4%. The proportion of coding regions was highest (nearly 70%) in the chloroplast genome (Table 1).

**Table 1:** Genome features of 12 *Nymphaea* chloroplast genomes

Genome features		*Nymphaea amazonum*	*Nymphaea conardii*	*Nymphaea glandulifera*	*Nymphaea gracilis*	*Nymphaea heudelotii*	*Nymphaea immutabilis*
Region Length	Complete chloroplast genome (bp)	159,186	159,57	159,093	159,797	159,850	160,042
	LSC (bp)	89,388	89,397	89,301	89,825	90,000	90,118
	SSC (bp)	19,472	19,514	19,466	19,576	19,504	19,594
	IR (bp)	25,163	25,173	25,163	25,198	25,173	25,165
GC Content	Total GC content (%)	39.1	39.1	39.1	39.2	39.2	39.1
	GC content in LSC (%)	37.8	37.8	37.8	37.8	37.8	37.7
	GC content in SSC (%)	34.2	34.2	34.2	34.4	34.4	34.4
	GC content in IR (%)	43.4	43.4	43.4	43.5	43.4	43.4
Gene Numbers	Number of genes [unique]	130 [113]	130 [113]	130 [113]	130 [113]	130 [113]	130 [113]
	Protein genes [unique]	85 [79]	85 [79]	85 [79]	85 [79]	85 [79]	85 [79]
	tRNA genes [unique]	37 [30]	37 [30]	37 [30]	37 [30]	37 [30]	37 [30]
	rRNA genes [unique]	8 [4]	8 [4]	8 [4]	8 [4]	8 [4]	8 [4]
	Duplicated genes in IR	16	16	16	16	16	16
Percentage of	Coding regions	69.61%	69.55%	69.66%	69.38%	69.35%	69.26%
	Intergenic sequences	30.39%	30.45%	30.34%	30.62%	30.65%	30.74%
	rRNA and tRNA genes	8.49%	8.48%	8.49%	8.45%	8.45%	8.44%
Accession numbers in GenBank		MW802262	MW802263	MW802264	MW802265	MW802266	MW802267

Genome features		*Nymphaea rudgeana*	*Nymphaea* sp. NY566	*Nymphaea* sp. NY590	*Nymphaea* sp. NY668	*Nymphaea* sp. NY701	*Nymphaea tenerinervia*
Region Length	Complete chloroplast genome (bp)	159,220	159,916	158,894	159,897	159,705	158,290
	LSC (bp)	89,605	89,960	89,132	89,947	89,822	88,499
	SSC (bp)	19,283	19,582	19,462	19,576	19,523	19,465
	IR (bp)	25,166	25,187	25,150	25,187	25,180	25,163
GC Content	Total GC content (%)	39.1	39.1	39.2	39.2	39.2	39.2
	GC content in LSC (%)	37.8	37.8	37.8	37.8	37.8	37.8
	GC content in SSC (%)	34.4	34.4	34.3	34.4	34.4	34.3
	GC content in IR (%)	43.4	43.4	43.4	43.4	43.4	43.4
Gene Numbers	Number of genes [unique]	130 [113]	130 [113]	130 [113]	130 [113]	131 [112]	130 [113]
	Protein genes [unique]	85 [79]	85 [79]	85 [79]	85 [79]	86 [78]	85 [79]
	tRNA genes [unique]	37 [30]	37 [30]	37 [30]	37 [30]	37 [30]	37 [30]
	rRNA genes [unique]	8 [4]	8 [4]	8 [4]	8 [4]	8 [4]	8 [4]
	Duplicated genes in IR	16	16	16	16	16	16
Percentage of	Coding regions	69.47%	69.35%	69.68%	69.35%	69.57%	70.00%
	Intergenic sequences	30.53%	30.65%	30.32%	30.65%	30.43%	30.00%
	rRNA and tRNA genes	8.48%	8.45%	8.50%	8.45%	8.48%	8.53%
Accession numbers in GenBank		MW802268	MW802269	MW802270	MW802271	MW802272	MW802273

According to the annotated results, considerable similarities in the type and number of genes were found between all 12 species of *Nymphaea*. The chloroplast genomes of most *Nymphaea* species had a total gene number of 130, except for *N.* sp. NY701, which had one more copy of *rps15*. The chloroplast genome of *Nymphaea* contained 113 non-repeating genes, including 79 protein-coding genes, 30 tRNA genes, and four rRNA genes. These genes can be roughly divided into three categories: chloroplast self-replication genes, photosynthesis-related genes, and other genes (Table 2). Introns were found in 12 genes (one intron: *trnA-*UGC*, trnG-*GCC*, trnI-*GAU*, trnK-*UUU*, trnV-*UAC*, rps12, rpl2, rpoC1, ndhA, ndhB, atpF*; two introns: *clpP, ycf3*). The 17 repeating genes were all located in the IR region, including four rRNA genes, seven tRNA genes, and six or seven protein-coding genes.

**Table 2:** Genes present in the chloroplast genome of *Nymphaea*

Category of genes	Group of genes	Gene name
Self-replication	rRNA genes	*rrn4.5*^*(a)*^*, rrn5*^*(a)*^*, rrn16*^*(a)*^*, rrn23*^*(a)*^
	tRNA genes	*trnA-*UGC^*(ab)*^*, trnC-*GCA*, trnD-*GUC*, trnE-*UUC*, trnF-*GAA*, trnfM-*CAU*, trnG-*UCC*, trnG-*GCC^*(b)*^*, trnH-*GUG*, trnI-*CAU^*(a)*^*, trnI-*GAU^(ab*)*^*, trnK-*UUU^*(b)*^*, trnL-*CAA^*(a)*^*, trnL-*UAA^*(b)*^*, trnL-*UAG*, trnM-*CAU*, trnN-*GUU^*(a)*^*, trnP-*UGG*, trnQ-*UUG*, trnR-*UCU*, trnR-*ACG^(^^*a)*^*, trnS-*GCU*, trnS-*GGA*, trnS-*UGA*, trnT-*GGU*, trnT-*UGU*, trnV-*GAC^*(a)*^*, trnV-*UAC^*(b)*^*, trnW-*CCA*, trnY-*GUA
	Small subunit of ribosomal proteins	*rps2, rps3, rps4, rps7*^*(a)*^*, rps8, rps11, rps12*^*(ab)*^*, rps14, rps15, rps16, rps18, rps19*
	Large subunit of ribosomal proteins	*rpl2*^*(ab)*^*, rpl14, rpl16, rpl20, rpl22, rpl23*^*(a)*^*, rpl32, rpl33, rpl36*
	DNA-dependent RNA polymerase	*rpoA, rpoB, rpoC1*^*(b)*^*, rpoC2*
Photosynthesis	Rubisco	*rbcL*
	Photosystem I	*psaA, psaB, psaC, psaI, psaJ*
	Photosystem II	*psbA, psbB, psbC, psbD, psbE, psbF, psbH, psbI, psbJ, psbK, psbL, psbM, psbN, psbT, psbZ*
	NADH oxidoreductase	*ndhA*^*(b)*^*, ndhB*^*(ab)*^*, ndhC, ndhD, ndhE, ndhF, ndhG, ndhH, ndhI, ndhJ, ndhK*
	Cytochrome b6/f complex	*petA, petB, petD, petG, petL, petN*
	ATP synthase	*atpA, atpB, atpE, atpF*^*(b)*^*, atpH, atpI*
Other genes	Maturase	*matK*
	Protease	*clpP*^*(c)*^
	Translational initiation factor	*infA*
	Subunit acetyl-CoA-carboxylase	*accD*
	c-Type cytochrome synthesis gene	*ccsA*
	Envelope membrane protein	*cemA*
	Conserved hypothetical chloroplast ORF	*ycf1, ycf2*^*(a)*^*, ycf3*^*(c)*^*, ycf4*

^(a)^Two gene copies in IRs; ^(b)^gene containing a single intron; ^(c)^gene containing two introns

### Codon usage and RNA editing sites analysis

**Fig. 2:**
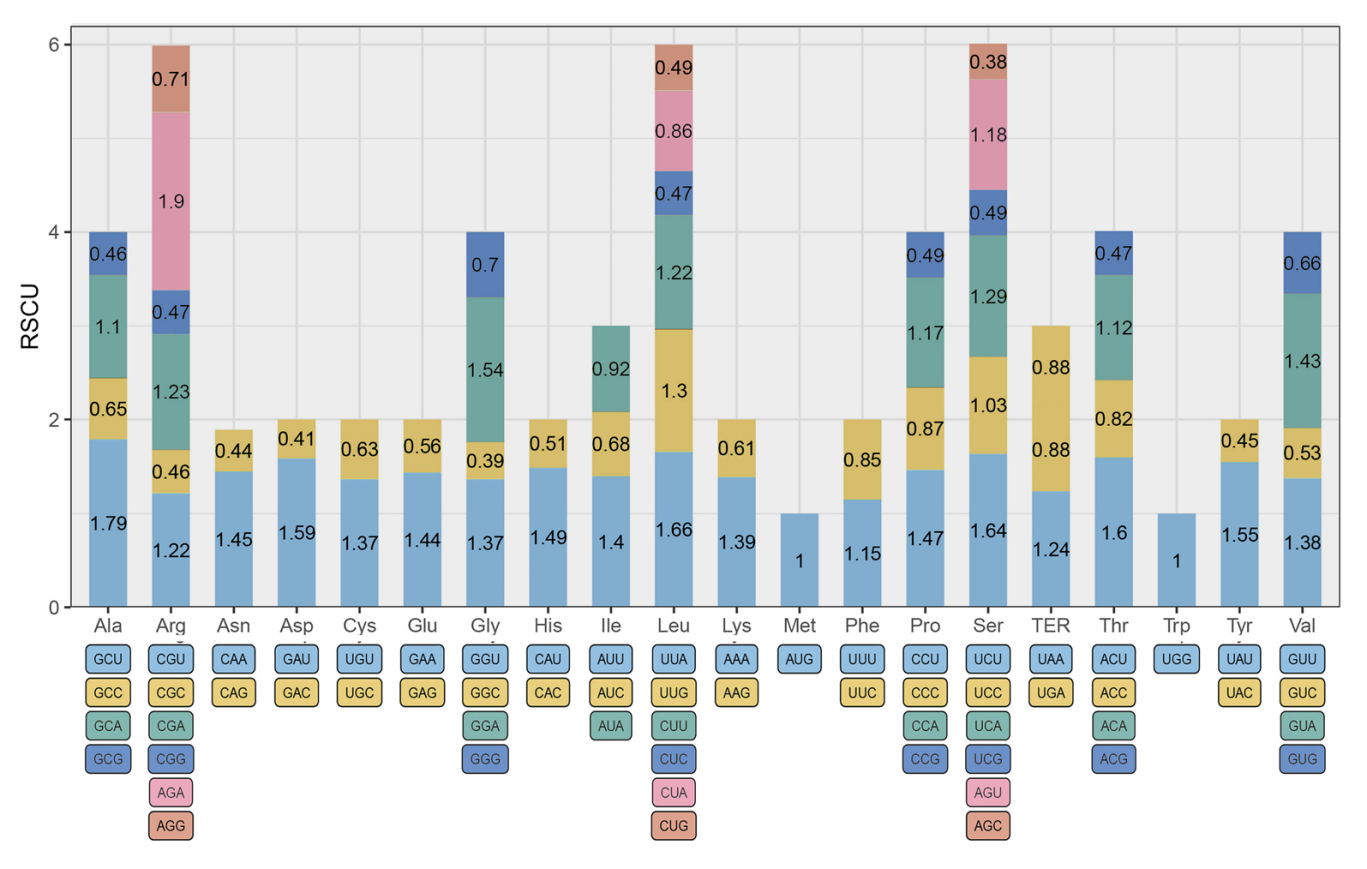
Bias in codon usage as shown by RSCU values with each amino acid. For the chloroplast genome of *N. immutabilis*, the codon usage for 20 amino acids, and the stop codons of all protein-coding genes identified are shown

Since *N. immutabilis* had the largest chloroplast genome, we used it as an example to calculate the codon usage bias and RSCU values of 84 CDS genes (Fig. 2; Supplementary Table S3). The results showed that among all the codons of *N. immutabilis*, 31 had an RSCU value greater than 1, indicating that these were preferred codons*.* Moreover, 30 codons ended with A/U. Among these codons, UUA, UCU, ACU, GCU, and AGA showed strong bias (RSCU ≥ 1.6). However, the RSCU values of codon AUG encoding methionine and codon UGG encoding tryptophan were both 1, indicating that there is no bias in terms of the usage of methionine and tryptophan in *N. immutabilis*. A total of 26,338 codons were found in these coding regions. The most common amino acids were leucine (2679 codons), isoleucine (2197 codons) and serine (2079 codons), while cysteine had the fewest codons (309).

RNA editing refers to the replacement, insertion, and deletion of nucleotides in the process of RNA maturation after transcription. We used the PREP suite to analyze the RNA editing sites of 26 protein-coding genes in the cp genome of *N. amazonum*. A total of 96 RNA editing sites were detected, most of which involved the conversion of serine to leucine (Supplementary Table S4). The most frequently edited genes were *rpoC2* (13 sites), *ndhA* (10 sites) and *ndhB* (9 sites). All the observed changes occurred at the first or second nucleotide site in each codon. Most RNA editing sites resulted in the conversion of polar amino acids to nonpolar amino acids, with the commonest conversion being serine to leucine or phenylalanine.

### SSRs and long repeats analysis

**Fig. 3:**
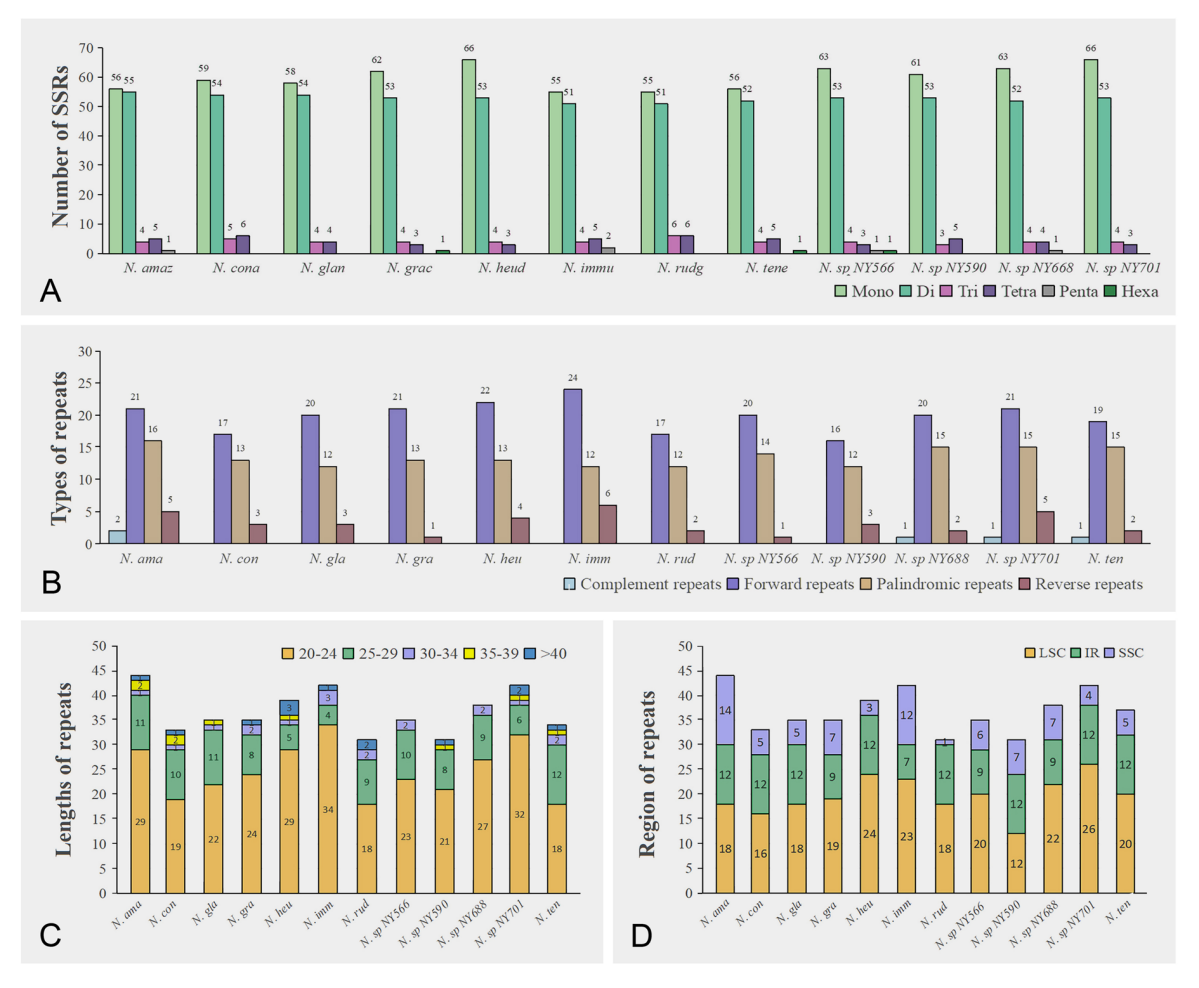
Analysis of repeats in 12 *Nymphaea* chloroplast genomes. **A** Number of SSRs. **B** Types of repeats. **C** Lengths of repeats. **D** Regions of repeats

Six types of SSR were measured, namely mononucleotide, dinucleotide, trinucleotide, tetranucleotide, pentanucleotide, and hexanucleotide repeats. A total of 1464 SSR markers, ranging in length from 8 to 183 bp, were detected in the chloroplast genomes of the 12 *Nymphaea* species. According to the data, the first four repeat contents were significant, while the number of pentanucleotide and hexanucleotide repeats was relatively low or even non-existent. The number of SSRs in the chloroplast genomes of the 12 species ranged from 117 (*N. immutabilis*) to 126 (*N. heudelotii* and *N.* sp. NY701) (Fig. 3A; Supplementary Table S5). The number of mononucleotide repeats was the largest, accounting for about 49.81% of the total number of SSRs. 89.72% of these were A/T repeats, and a few tandem cytosine and guanine were found. The dinucleotide repeats were the second most common; 59.46% of these were AT/AT repeats, and the rest were AG/CT and AC/GT repeats. The number of trinucleotide repeats accounted for 3.42% of all SSRs. There were three types of trinucleotide repeats (AAG/CTT, AAT/ATT, and AGG/CCT) in all species except for *N.* sp. NY590. There were six different types of tetranucleotide repeats of these the chloroplast genomes of all 12 species all had AAAG/CTTT and AATC/ATTG repeats, while the other four tetranucleotide repeats were rare or found in only a few species.

The number of long repeats detected in this experiment ranged from 20 (*N. heudelotii*) to 49 (*N. amazonum*), and the types and numbers of long repeats varied widely from species to species (Fig. 3B). The number of forward and palindromic repeats was higher than that of reverse and complementary repeats. Indeed, only four species (*N. amazonum, N.* sp. NY668, *N.* sp. NY701, and *N. tenerinervia*) contained complementary repeats. Moreover, in four types of repeats, the long repeats were the most abundant, and the complementary repeats were the least abundant. The length of repeats ranged from 20 to 24 bp (Fig. 3C). The distribution of repetitive sequences was also region-specific, with the largest number of repetitive sequences in the LSC region (Fig. 3D).

**Fig. 4:**
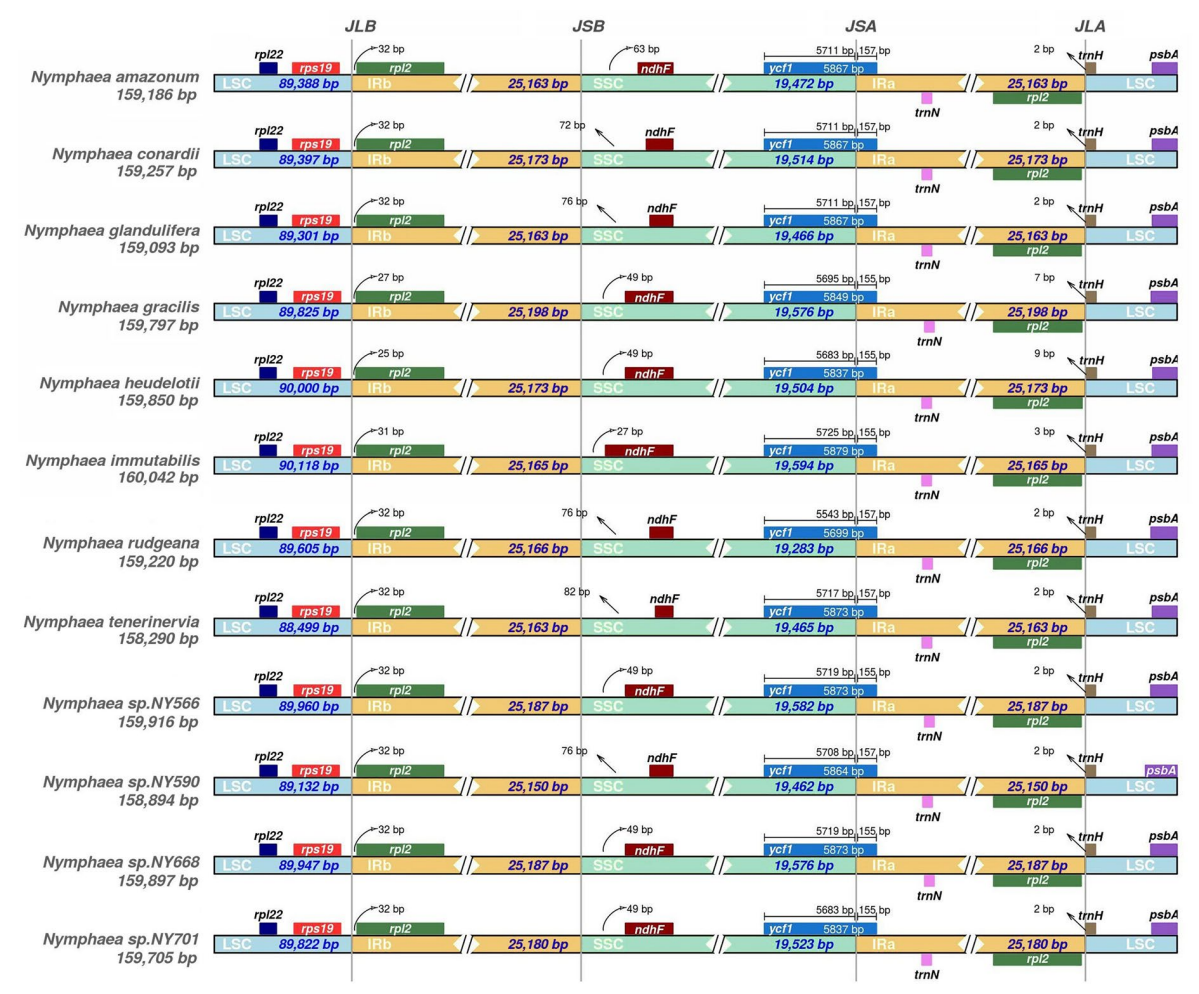
Junctions in 12 *Nymphaea* chloroplast genomes were compared for LSC, IRb, SSC, and IRa. The LSC/IRb, IRb/SSC, SSC/IRa, and IRa/LSC junctions are represented by loci JLB, JSB, JSA, and JLA, respectively

The chloroplast genome is made up of four regions (LSC, SSC, IRa, and IRb), thus forming four boundaries. In genome evolution, the differences between species of the same genus are often associated with the expansion and contraction of IR regions (Wang et al. 2008). We conducted a comprehensive comparative analysis of four junctions (JLA, JLB, JSA, and JSB) between two IR regions (IRa and IRb) and two single-copy regions (LSC and SSC) in 12 *Nymphaea* species (Fig. 4). Although the genomes of IR regions in the 12 *Nymphaea* species were similar in size, ranging from 25,150 to 25,198 bp, some expansion and contraction could be observed. The JSA boundary was spanned by the *ycf1* gene, with a length difference of less than 2 bp between different species. The *ndhF* gene was located near the JSB boundary, with the *ndhF* of *N. immutabilis* being the closest to the JSB line (27 bp distant) and that of *N. tenerinervia* being the farthest (82 bp distant). The *rpl2* and *trnH* genes were located in the JLA boundary, which was spanned by the *trnH* gene. *trnH* was mainly located in the LSC region and extended to the IRa region by 2–9 bp. The *rps19* and *rpl2* genes were located in the JLB boundary, where the *rpl2* was 25–32 bp away from the junction. Expansion and contraction of the IR regions were presumably the main reason for the variation in chloroplast genome size, which is consistent with the previous reports (Asaf et al. 2016; Li et al. 2018).

### Comparative analysis

**Fig. 5:**
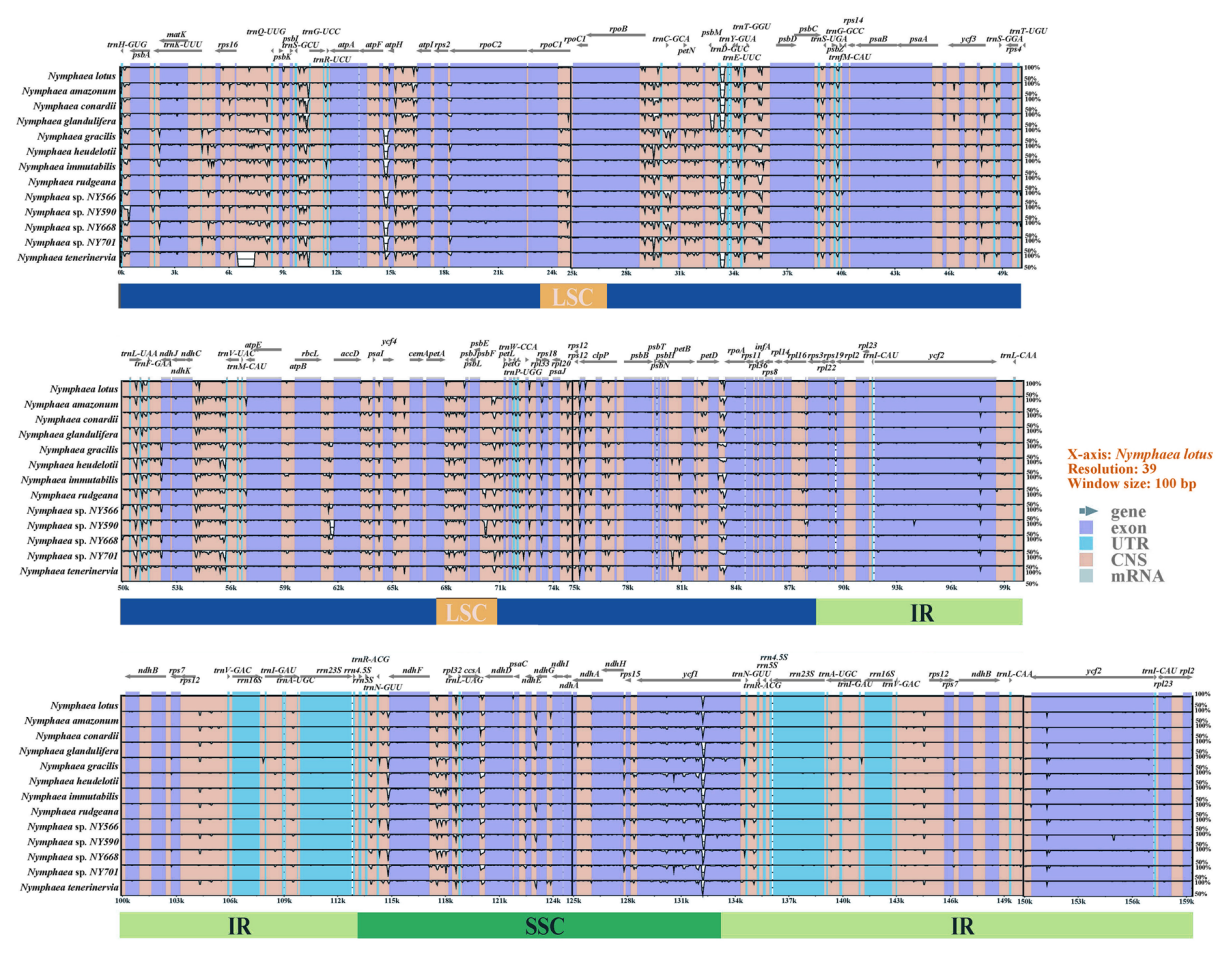
Comparison of chloroplast genomes of 12 *Nymphaea* species. The direction of the gene is indicated by the gray arrow above the comparison. Exons are shown in dark blue, untranslated regions (UTR) in light blue, and conserved non-coding sequences (CNS) in pink. The percentage consistency on the Y-axis ranges from 50% to 100%

The chloroplast genomes were highly conserved in terms of structure and gene sequence (Fig. 5). The variation in the IR region was considerably lower than that of the LSC and SSC regions. Moreover, the difference in the non-coding sequence region was greater than that in the coding region, as the former had more mutations than the latter (Widmer and Baltisberger 1999). Regions with significant variation in the chloroplast genome of *Nymphaea* generally appeared in the intergenic spacer regions (IGS), such as between *trnH-*GUG and *psbA*, *trnK-*UUU and *rps16*, *rps16* and *trnQ-*UUG, *trnS-*GCU and t*rnG-*UCC, *atpF* and *atpH*, *atpH* and *atpI*, rpoB and *trnC-*GCA, *trnC-*GCA and *petN*, *petN* and *psbM*, *psbM* and *trnD-*GUC, *trnD-*GUC and *trnY-*GUA, *trnT-*GGU and *psbD*, and *psbD* and *rpoA*. The results also showed that the protein-coding sequence regions were highly conserved, especially the rRNA gene, with almost no variation observed.

### Select pressure and nucleotide diversity analysis

Non-synonymous substitution (Ka), synonymous substitution (Ks), and their ratio (Ka/Ks) are important indicators for understanding the direction of evolution and selection (Li et al. 2009). Therefore, we looked at these variables in 79 protein-coding genes (PCGs) (Supplementary Table S6). Ka/Ks > 1 indicates positive selection, Ka/Ks < 1 indicates negative selection, and Ka/Ks≈1 indicates neutral evolution (Li et al. 2009). Compared with other genes, the Ka/Ks ratios of photosystem I and photosystem II genes were either equal to or close to 0. This indicated that these genes were highly conserved and showed an elevated level of purification selection. The genes with positive selection were *atpF* (except *N.* sp*.* NY590), *clpP* (*N. amazonum*, *N. rudgeana, N. tenerinervia*, and *N.* sp. NY590), *ndhA* (*N. amazonum, N. conardii, N. glandulifera, N. tenerinervia,* and *N.* sp. NY590), *ycf2* (*N. gracilis, N. heudelotii, N*. sp. NY566, *N.* sp. NY668, and *N.* sp. NY701), and *ycf3* (*N. heudelotii, N. immutabilis, N.* sp. NY566, *N.* sp. NY668, and *N.* sp. NY701). The Ka/Ks ratio of the *atpF* gene was greater than 1.35 in all *Nymphaea* species except *N.* sp. NY590. This suggested the presence of beneficial mutations and rapid development of the *atpF* gene in these species. On the other hand, the Ka/Ks ratios of the *atpE*, *clpP*, *ndhA*, *ndhD*, *ndhK*, *petB,* and *ycf3* genes in some species were equal to or close to 1, indicating that these genes had neutral evolution.

The nucleotide diversity value (Pi value) reflects the base diversity level of the population genome. The Pi value was used to analyze base polymorphism. The lower the level of polymorphism, the higher the degree of selection. To fully understand the differences between sequences, we performed slicing window analysis to visualize the nucleotide variation values. A total of 17 regions with high divergence values (Pi > 0.07) were identified and designated as hypervariable regions. Among the 17 highly variable regions, 10 were located in the LSC region, seven in the SSC region, and none in the IR regions. It can also be clearly seen from Supplementary Fig. S2 that the SSC and LSC regions were more distinctly different than IR regions, and the Pi value of the IR region was considerably lower.

### Phylogenetic analysis and divergence time estimation of Nymphaeales

**Fig. 6:**
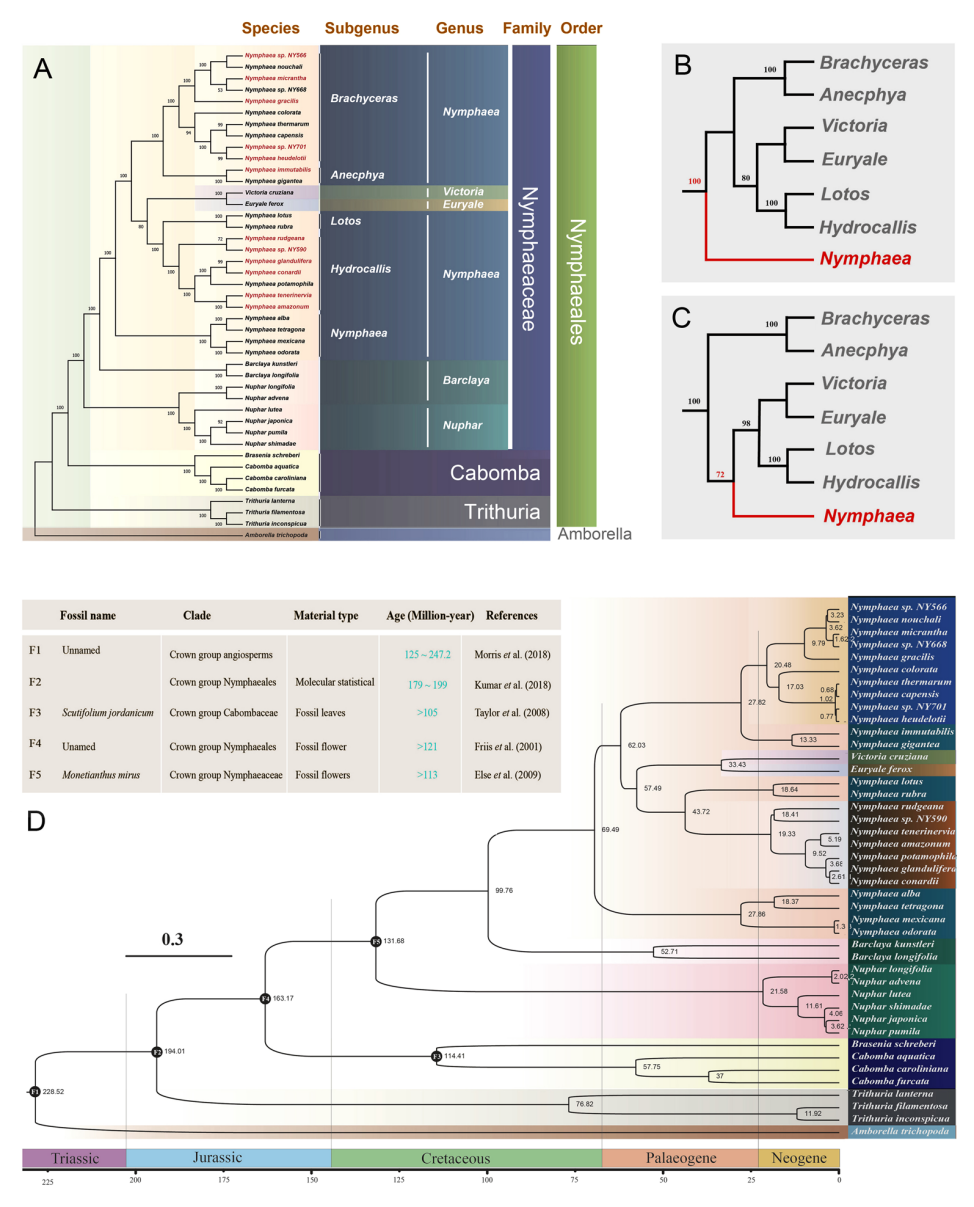
Phylogenetic relationships and divergence times of the Nymphaeales. **A** Phylogenetic tree constructed using 78 protein-coding genes. **B** Phylogenetic tree constructed from protein-coding genes. **C** Phylogenetic tree constructed from complete chloroplast genes. **D** A time evolutionary tree constrained by a relaxed molecular clock

To infer the taxonomy and phylogeny of Nymphaeales, we constructed two phylogenetic trees using 79 CDS genes and the complete chloroplast genomes, respectively. Our results are consistent with the view that Nymphaeales includes three families, namely Hydatellaceae, Cabombaceae, and Nymphaeaceae (Fig. 6A). There are five subgenera in *Nymphaea*, namely *Brachyceras*, *Anecphya*, *Lotos*, *Hydrocallis*, and *Nymphaea*. Twelve species with newly sequenced genomes were placed in the subgenera *Brachyceras* (five species)*, Anecphya* (one species)*,* and *Hydrocallis* (six species). Notably, the phylogenetic tree indicated that *Victoria* and *Euryale* were placed within *Nymphaea*, as sister taxa to the subgenera of *Lotos* and *Hydrocallis*. In addition, there were some differences between the two trees, such as sister relationships and bootstrap values, and especially the position of the subgenus *Nymphaea*. The subgenus *Nymphaea* was sister to a clade comprising all other subgenera plus *Victoria* and *Euryale* in the 79 CDS tree (Fig. 6B), whereas it was sister to a clade comprising *Hydrocallis, Lotos, Euryale* and *Victoria* in the complete chloroplast genome tree (Fig. 6C).

To estimate the divergence times of taxa within the order Nymphaeales, a time tree was constructed using a relaxed molecular clock calibrated using one molecular-based and four fossil-based ages (Fig. 6D). The result of the time estimation had the following noteworthy points (Fig. 6D; Supplementary Table S7): (a) *Amborella trichopoda*, considered to be the oldest angiosperm, diverged around 228.52 million years ago (Ma). (b) Nymphaeales originated about 200 million years ago, Hydatellaceae about 194.01 Ma, Cabombaceae about 163.17 Ma, and Nymphaeaceae about 131.68 Ma. (c) *Nymphaea* and *Barclaya* diverged about 99.76 Ma. (d) Diversification of the five subgenera of *Nymphaea* occurred 69.49 Ma, with the *Nymphaea* being the oldest subgenus having originated about 66.97 Ma and subgenera *Brachyceras* and *Anecphya* being the youngest having originated about 27.82 Ma. It is also noteworthy that *Victoria* and *Euryale*, as two genera of Nymphaeaceae, diverged about 57.49 Ma, meaning that they have a later origin than the subgenus *Nymphaea.*

## Discussion

### Plastome features

In most higher plants, plastids are maternally inherited and exhibit highly conserved structures, showing little recombination (Birky 1995). With the development of sequencing technology, several comparative studies have revealed that chloroplast genomes exhibit similarities in terms of genome size, GC content, and gene type (Song et al. 2022a; Yang et al. 2022). For example: (i) the chloroplast genome is a circular double-stranded DNA molecule, ranging in size between 120 and 180 kb; (ii) the GC content in the IR region is relatively higher than that in the LSC and SSC regions; (iii) the number of genes is estimated to be around 120–140, including a protein-coding genome of 70–80 genes, around 37 tRNA genes, and approximately eight rRNA genes. A previous study found gene loss in the chloroplast genomes of *Nymphaea*, such as *ycf1*, *ndhF*, and *rpoC2* (Sun et al. 2021)*.* However, no genes were lost in our 12 chloroplast genomes of *Nymphaea*, all of which contained 113 unique genes. These genes formed a circle, with a pair of inverted repeat sequences dividing it into four parts (Sato et al. 1999). We found that the high GC content of the IR region was attributed to the abundance of tRNA and rRNA genes, which were predominantly composed of GC base pairs (Doorduin et al. 2011).

Relative synonymous codon usage (RSCU) is an indicator used to evaluate the preferences for evaluating 59 synonymous codons to study variations in synonymous codon use between genes. The high relative similarity in codon usage among different species suggested that a similar environmental selection may have been experienced (Yang et al. 2014). In the genome, we found a tendency to use codon endings with A/T in high-use codons (RSCU > 1), which was also frequently observed in angiosperms (Mehmood et al. 2020). Our analysis revealed few differences in the RSCU of water lily chloroplast genes, suggesting that codon usage was conserved (Liu and Xue 2005; Zhou et al. 2008). It has been shown that RNA editing usually occurs at the first or second base of the codon, which tends to shift amino acids from hydrophilic to hydrophobic and from polar to non-polar, resulting in an increase in the hydrophobicity of the protein (Chen et al. 2011; Han et al. 2022). Our data support this view, with a large amount of serine (hydrophilic) converted to leucine (hydrophobic) and phenylalanine (hydrophobic).

Repeated sequences in the chloroplast genome may have the potential to promote gene rearrangement and recombination (Zhou et al. 2019). Among the 12 *Nymphaea* species, the most common type of repeat was mononucleotide, and the majority of these repeats were AT-enriched. This observation is consistent with the finding that chloroplast SSRs typically consist of short polyA or polyT repeats (Ye et al. 2018). It is noteworthy that the repetitive sequences rarely contained G-C bases, possibly due to the fact that G-C bases form three hydrogen bonds, which makes them more resistant to disruption and less conducive to genetic recombination (Li et al. 2021b). We detected long repeats in nine *Nymphaea* species, most of which were located in the LSC region. Repeat sequences play a role in various processes, such as gene activity, genome organization, DNA replication, recombination, and repair (He et al. 2018).

### Comparative genomics

In angiosperms, the border positions of IR/LSC are not conserved, having frequent contractions and expansions (He et al. 2018), which is the main reason for the variation of chloroplast genome length (Chumley et al. 2006). Furthermore, the expansion and contraction of the region are associated with the structure of the chloroplast, leading to changes in gene number and position (Yang et al. 2022). For example, an alteration of the IR region makes an extra copy of the *rps19* gene in *Musa* chloroplasts (Song et al. 2022b). In the present study, the IR region also showed expansion and contraction, with the presence of an additional copy of the *rps15* gene in *N.* sp. NY701 and a change in the distance between the *ndhF* gene and the JSB boundary. In addition, previous studies found the absence of the *ndhF* gene in *N. odorata* and of the *ycf1* gene in *N. tetragona* due to changes in the IR region (Sun et al. 2021). However, gene loss was not found in the present study, which may be related to the selective pressure of the habitats of the 12 *Nymphaea* species (Cheng et al. 2021).

Chloroplasts were relatively stable in nucleotide content and highly conserved in gene structure, but there were hotspots of variation. These hotspots with relatively high mutation rates can be used as DNA barcoding for plant identification (Ge et al. 2019; Kuang et al. 2011). In our study, the comparison of 12 *Nymphaea* species revealed higher variation in the non-coding regions than in the coding regions. Therefore, the speed of molecular evolution in non-coding regions provide a good basis for phylogenetic inference (Shi et al. 2022; Zhu and Ge 2005). In addition, the nucleotide diversity results showed that most of the highly variable regions were located in the SC, and the IR regions were highly conserved (Smith 2015). By comparing the chloroplast genome sequences, it is possible to find similarities and homologies between species and predict evolutionary relationships between sequences (Wu and Chaw 2015).

The ratio of synonymous (Ks) and non-synonymous (Ka) substitutions is important for inferring evolutionary rates and for understanding adaptive development among species (Fay and Wu 2003). In chloroplast protein-coding genes, synonymous substitutions are usually more frequent than nonsynonymous substitutions, with ratios of KA/KS < 1 (Abdullah et al. 2020). The Maturases, Ubiquinol Cytochrome C reductase, and Cytochrome C biogenesis genes also exhibit Ka/Ks ratios below 1, indicating a negative selection (Cheng et al. 2021). In the present study, most Ks were much higher than Ka, implying a relatively slow evolution of *Nymphaea* species. However, the Ka/Ks ratios of *atpF*, *ndhA*, *clpP*, *ycf2*, and *ycf3* were greater than 1, meaning that these genes had undergone positive selection by the environment. The six ATP synthase genes, *atpA*, *atpB*, *atpE*, *atpH*, and *atpI* had very low Ka/Ks values, and only *atpF* had a Ka/Ks value above 1. Neutral theory shows that most of the mutations at the molecular level, such as nucleotide substitution in gene spacers and introns, and the pseudogenes that are not translated into proteins, are neutral or nearly neutral, meaning they were not subject to natural selection (Fay and Wu 2001).

### Phylogeny and evolution

The genetic and intergenic regions of the chloroplast genome have different rates of molecular evolution, which provide differing genetic variation for phylogenetic studies (Clegg et al. 1994; Jian et al. 2008). Earlier phylogenetic analyses of Nymphaeales were limited to the use of one or a few marker fragments from either the plastid or nuclear genome (Biswal et al. 2012; Les et al. 1991). With the rapidly increasing number of sequenced plastid genomes, more recent studies have used complete chloroplast genomes to analyze evolutionary relationships, but few systematic studies have been conducted on the basal taxa of flowering plants (He et al. 2018; Sun et al. 2021). Here, we used the complete chloroplast genomes and coding regions of 43 species to construct comprehensive phylogenetic relationships within Nymphaeales and estimate divergence times for early angiosperms. In previous studies, the relationship between *Cabomba* and *Brasenia* was controversial (Sokoloff et al. 2008; Sun et al. 2021). Some studies suggested that the two genera should be part of the Nymphaeaceae, while others reported that they could be members of Cabombaceae (Biswal et al. 2012; Dkhar et al. 2012; Löhne et al. 2007). The findings of the present study strongly supported the latter, implying that members of the order Nymphaeales are clearly divided into three families: Hydatellaceae, Cabombaceae, and Nymphaeaceae.

Zhang et al. (2020) concluded that *Victoria* and *Euryale* should be divided into separate genera based on phylogenetic analyses of nuclear genome data. However, *Victoria*, *Euryale,* and *Nymphaea* formed a clade in our phylogenetic tree. Previous studies suggested that the inconsistency between the positions of the *Euryale* + *Victoria* clade in the chloroplast tree and nuclear gene tree might be caused by chloroplast capture (Liu et al. 2017; Sun et al. 2021). Our results indicated that *Victoria* and *Euryale* should be assigned to the genus *Nymphaea* for the following two reasons: (a) the behavior of chloroplast capture has occurred only rarely throughout evolutionary time and has not been found in species that are closely related (Kawabe et al. 2018; Yang et al. 2021); and (b) chloroplast genomes are more stable when maternally inherited gene (Kumar et al. 2004). Conard (1905) was the first to divide *Nymphaea* into five subgenera, and this conclusion was further supported by the molecular findings of Borsch et al. (2007). Our phylogenetic tree also support the division of *Nymphaea* into five subgenera: *Brachyceras*, *Anecphya*, *Lotos*, *Hydrocallis*, and *Nymphaea*. However, we found that *Brachyceras* and *Anecphya* form one branch, and *Lotos* and *Hydrocallis* form another branch in the evolutionary tree. As a result, it was more parsimonious to conclude that *Nymphaea* should be divided into three subgenera: *Brachyceras*-*Anecphya*, *Lotos*-*Hydrocallis,* and *Nymphaea,* which consistent with previous studies (Dkhar et al. 2012; Löhne et al. 2007).

Angiosperms evolved a diversity of species during a relatively short geological period—Darwin’s ‘abominable mystery’ (Shi et al. 2022). In recent years, molecular time trees have been used to estimate the divergence times within angiosperms (Li et al. 2019; Wu et al. 2014; Zeng et al. 2014). We provide here for the first time a near-complete temporal framework for the evolution of Nymphaeales above the generic level. Although previous studies have acknowledged a significant gap in the fossil record of the angiosperm stem lineage (Bell et al. 2010; Magallón 2010), there is no compelling argument supporting a specific maximum age constraint for the crown node of angiosperms (Massoni et al. 2015). We restricted ourselves to conservative fossil age constraints based on the timescale of early land plant evolution (Morris et al. 2018). We used the chloroplast genome to estimate the time of divergence for angiosperms at about 228 Ma, the time of divergence for the Nymphaeales at about 194 Ma (crown group), the time of divergence for the Nymphaeaceae at about 131 Ma, and the time of divergence for the genus *Nymphaea* at about 69 Ma. Our divergence times were a little later than those inferred for the nuclear genome, which may be attributed to the more conserved chloroplast genome (Dong et al. 2013; Zhang et al. 2020). In general, new age estimates for species, families and orders of angiosperms are compatible with the putative fossil record attributed to each of these taxa (Foster and Ho 2017; Li et al. 2019; Ran et al. 2018). Molecular time estimation should be seen as an attempt to reduce the range of the most likely ages for nodes constrained by the age of reliable fossil records securely placed and dated (Kumar et al. 2017) and as a method to evaluate the probable ages of nodes for which there is no direct fossil record (Bouckaert et al. 2014; Puttick 2019). Therefore, we should take into account the ambiguity of our current knowledge since molecular dating approaches cannot provide unambiguous ages except for particularly fossil-rich clades (Massoni et al. 2015). Future studies of molecular dating will likely use additional fossils, which could revise several estimates supported in the current study and decrease the size of the credibility intervals.

## Conclusions

Here, we sequenced and compared the chloroplast genomes of 12 *Nymphaea* species for the first time. The results showed that three amino acids (leucine, isoleucine, and serine) had a specific usage preference in *N. immutabilis* and that RNA editing sites resulted in the conversion of polar to non-polar amino acids. Contraction and expansion of the IR regions led to genome size differences, gene duplications, and deletions. Regions with high variations in the chloroplast genome of *Nymphaea* were generally in the intergenic spacer areas. The nucleotide diversity in the LSC and SSC regions was much higher than that in the IR region. The Ka/Ks ratios of the *atpF*, *ndhA*, *clpP*, *ycf2*, and *ycf3* genes were greater than 1, meaning that these genes had undergone positive selection by the environment. The results of the phylogenetic analysis and estimated divergence time will be helpful for future evolutionary studies of the basal taxa of angiosperms.

### Supplementary Information

Below is the link to the electronic supplementary material.

Figure S1: The results of mapping the reads to the assembled *Nymphaea* complete chloroplast genome sequences (PDF 1481 KB)

Figure S2: Sliding window analysis of 12 *Nymphaea* chloroplast genomes (PDF 262 KB)

Table S1: Information on raw sequencing data (XLSX 11 KB)

Table S2: Information on the 43 species in the phylogenetic tree (XLSX 13 KB)

Table S3: codon usage details (XLSX 12 KB)

Table S4: RNA editing sites raw data (XLSX 17 KB)

Table S5: Details of microsatellite structures (XLSX 12 KB)

Table S6: Value of Ka/Ks in 12 *Nymphaea* species (XLSX 30 KB)

Table S7: Time estimation tree with credibility intervals (XLSX 11 KB)

## Data Availability

The data that support the findings of this study are openly available in the GenBank database at https://www.ncbi.nlm.nih.gov/, under accession number [MW802262-MW802273].
