# Supplementary material for: Comparative analysis of 12 water lily plastid genomes reveals genomic divergence and evolutionary relationships in early flowering plants
Source: Mar Life Sci Technol. 2024 Aug 15;6(3):425–41. doi: 10.1007/s42995-024-00242-0 (PMC11358372; doi:10.1007/s42995-024-00242-0)

Supplementary Fig.1: The results of mapping the reads to the assembled *Nymphaea* complete chloroplast genome sequences

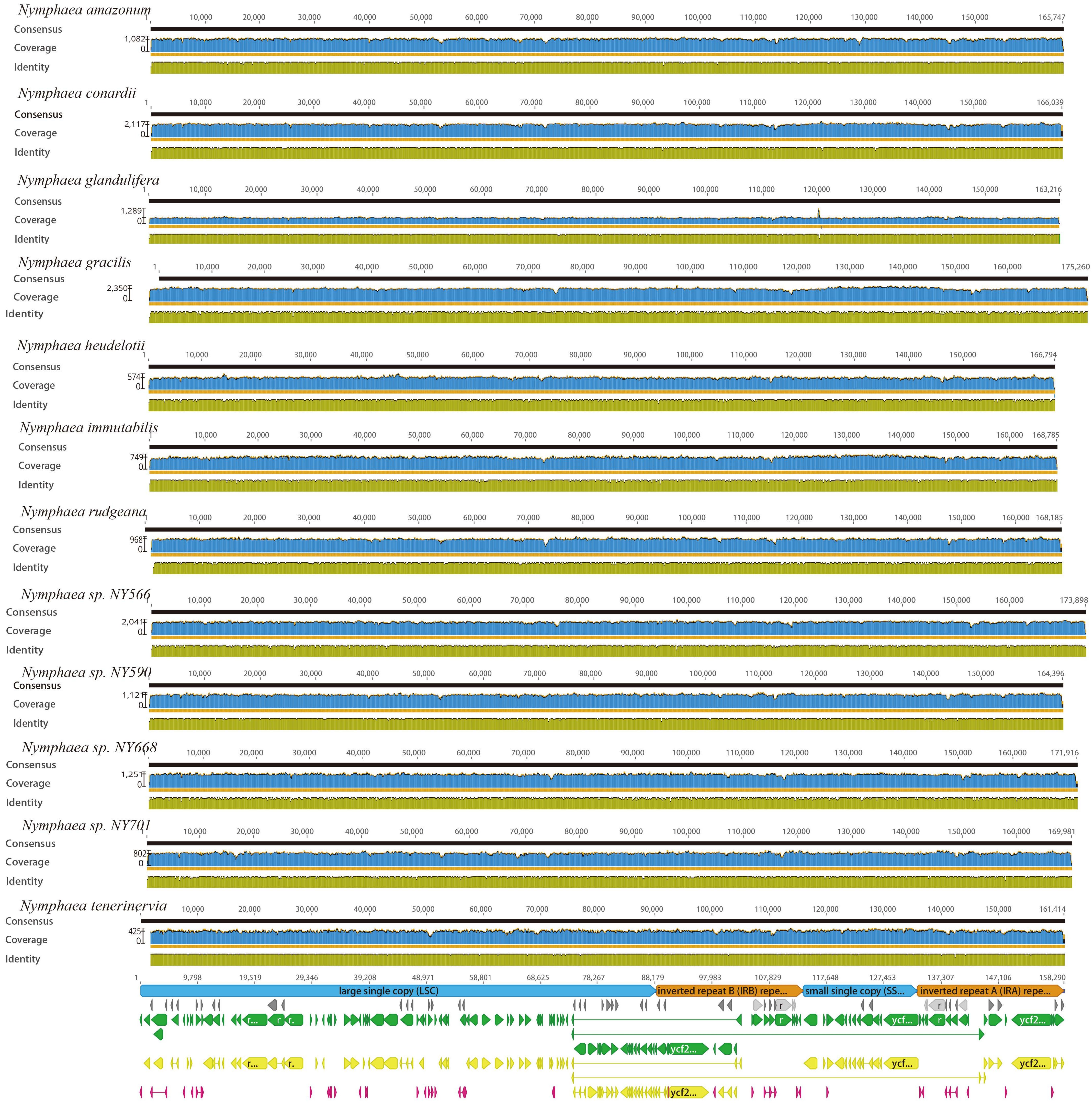

Supplement: Supplementary file 1 — Figure S1: The results of mapping the reads to the assembled Nymphaea complete chloroplast genome sequences (PDF 1481 KB) [file 42995_2024_242_MOESM1_ESM.pdf]
