# Supplementary figures and images for: Comparative analysis of 12 water lily plastid genomes reveals genomic divergence and evolutionary relationships in early flowering plants
Source: Mar Life Sci Technol. 2024 Aug 15;6(3):425–41. doi: 10.1007/s42995-024-00242-0 (PMC11358372; doi:10.1007/s42995-024-00242-0)

Supplementary Fig.2: Sliding window analysis of 12 *Nymphaea* chloroplast genomes

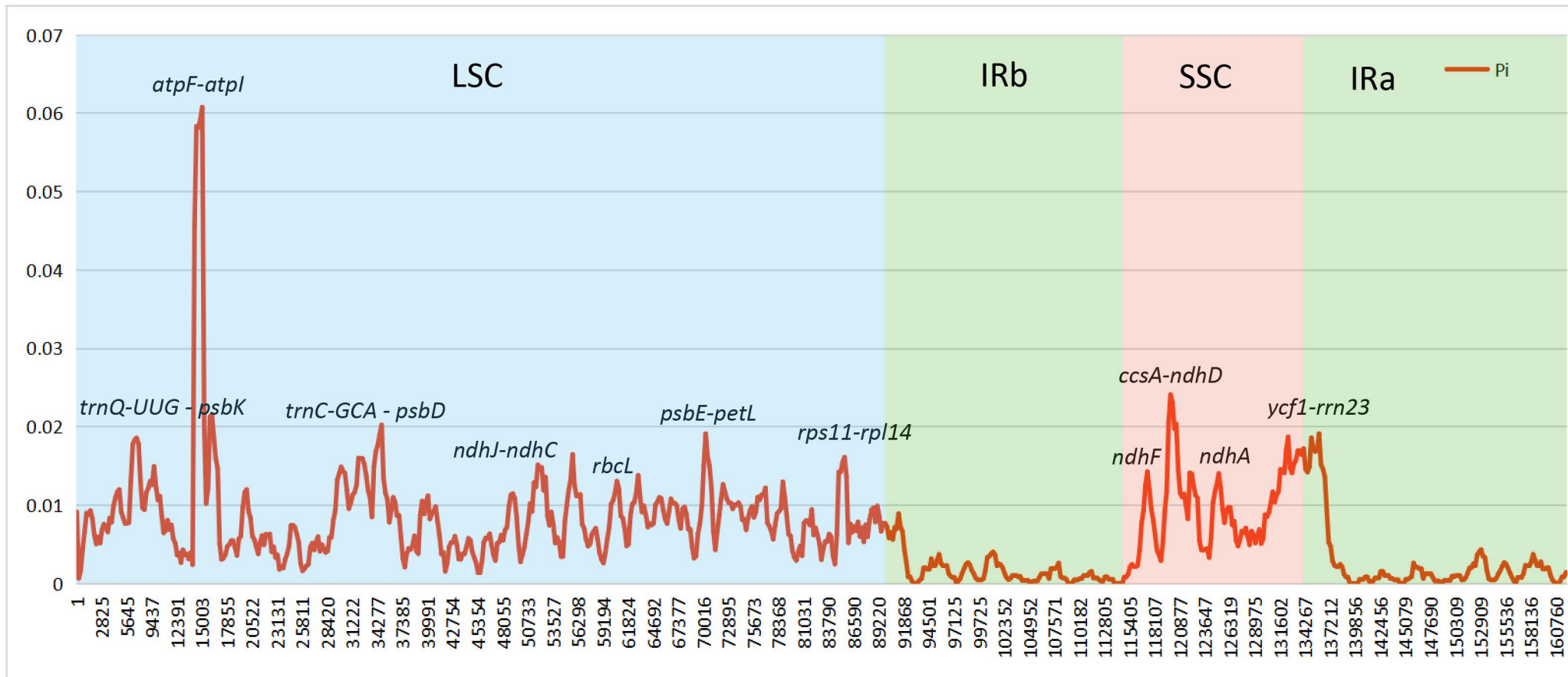

Supplement: Supplementary file 2 — Figure S2: Sliding window analysis of 12 Nymphaea chloroplast genomes (PDF 262 KB) [file 42995_2024_242_MOESM2_ESM.pdf]
